# Anti-Inflammatory Activity of *Ferula assafoetida* Oleo-Gum-Resin (Asafoetida) against TNF-*α*-Stimulated Human Umbilical Vein Endothelial Cells (HUVECs)

**DOI:** 10.1155/2022/5171525

**Published:** 2022-08-31

**Authors:** Leila Mobasheri, Mohsen Khorashadizadeh, Hossein Safarpour, Maryam Mohammadi, Gholamreza Anani Sarab, Vahid Reza Askari

**Affiliations:** ^1^Student Research Committee, Birjand University of Medical Sciences, Birjand, Iran; ^2^Department of Medical Immunology, Faculty of Medicine, Birjand University of Medical Sciences, Birjand, Iran; ^3^Cellular and Molecular Research Center, Birjand University of Medical Sciences, Birjand, Iran; ^4^Department of Medical Biotechnology, Faculty of Medicine, Birjand University of Medical Sciences, Birjand, Iran; ^5^Department of Medical Immunology, Faculty of Medicine, Shahed University of Medical Sciences, Tehran, Iran; ^6^Applied Biomedical Research Center, Mashhad University of Medical Sciences, Mashhad, Iran; ^7^Department of Pharmaceutical Sciences in Persian Medicine, School of Persian and Complementary Medicine, Mashhad University of Medical Sciences, Mashhad, Iran; ^8^International UNESCO Center for Health-Related Basic Sciences and Human Nutrition, Mashhad University of Medical Sciences, Mashhad, Iran

## Abstract

Inflammation is the body's biological reaction to endogenous and exogenous stimuli. Recent studies have demonstrated several anti-inflammatory properties of *Ferula* species. In this paper, we decided to study the anti-inflammatory effect of ethanolic extract of *Ferula assafoetida* oleo-gum-resin (asafoetida) against TNF-*α*-stimulated human umbilical vein endothelial cells (HUVECs). HUVECs were cultured in a flat-bottom plate and then treated with ethanolic extract of asafoetida (EEA, 0-500 *μ*g/ml) and TNF-*α* (0-100 ng/ml) for 24 h. We used the MTT test to assess cell survival. In addition, the LC-MS analysis was performed to determine the active substances. HUVECs were pretreated with EEA and then induced by TNF-*α*. Intracellular reactive oxygen species (ROS) and adhesion of peripheral blood mononuclear cells (PBMCs) to HUVECs were evaluated with DCFH-DA and CFSE fluorescent probes, respectively. Gene expression of intercellular cell adhesion molecule-1 (ICAM-1), vascular cell adhesion molecule 1 (VCAM-1), and E-selectin and surface expression of ICAM-1 protein were measured using real-time PCR and flow cytometry methods, respectively. While TNF-*α* significantly increased intracellular ROS formation and PBMC adhesion to TNF-*α*-induced HUVECs, the pretreatment of HUVECs with EEA (125 and 250 *μ*g/ml) significantly reduced the parameters. In addition, EEA pretreatment decreased TNF-*α*-induced mRNA expression of VCAM-1 and surface protein expression of ICAM-1 in the target cells. Taken together, the results indicated that EEA prevented ROS generation, triggered by TNF-*α*, and inhibited the expression of VCAM-1 and ICAM-1, leading to reduced PBMC adhesion. These findings suggest that EEA can probably have anti-inflammatory properties.

## 1. Introduction

Inflammation is the body's protective response against interior or foreign stimulators, which causes pain and tissue damage. When inflammation becomes chronic, it can lead to various conditions, including asthma, allergic reactions, cancer, cardiovascular diseases, thrombosis, and autoimmune disorders [1, 2].

Endothelial cells (E.C.s) are the essential modulators in vascular inflammation, and their dysfunction is associated with the induction and expansion of many chronic inflammatory problems [3]. Activation of E.C.s at the inflammatory site leads to the upregulation of endothelial cell adhesion molecules (ECAMs), which include vascular cell adhesion molecule 1 (VCAM-1), intercellular adhesion molecule 1 (ICAM-1), and E-selectin (CD62E) [4, 5]. Also, leukocyte migration following ECAM expression causes tissue and organ dysfunction by secreting inflammatory cytokines and chemokines, including tumour necrosis factor-*α* (TNF-*α*) and interleukin- (I.L.-) 1*β*, 6, and 8 [6, 7]. Furthermore, there is considerable evidence that TNF-*α* can trigger oxidative stress via increasing reactive oxygen species (ROS). ROS is reduced metabolites of oxygen that keep their oxidising abilities. Studies show that the expression quantity of VCAM-1 and ICAM-1 are directly connected to intracellular ROS synthesis [5, 8–10]. Therefore, suppression of ROS generation and ECAM expression can be a helpful medicinal approach for controlling vasculitis [11].

Corticosteroids and nonsteroidal anti-inflammatory drugs (NSAIDs) are common treatments that prevent chronic inflammation by reducing the production and secretion of TNF-*α*, IL-1*β*, ROS, and nitric oxide (NO) from the inflammatory cells [12]. However, patients may be affected by adverse effects of corticosteroids and NSAIDs, such as stomach pain and ulcers, kidney failure, Cushing's syndrome, high blood pressure, diabetes, fragile bones, and vulnerability to infections [13, 14]. Therefore, new compounds with minimal adverse effects tend to be detected and launched.

Herbal medicines and extracts have been commonly used for many years due to their possessing active compounds that might provide a valuable platform for drug discovery [2, 15]. For example, *assafoetida* is an oleo-gum-resin derived by cutting the stems and roots of the different *Ferula* species, such as *F. assafoetida L.* (as the main source), *F. foetida*, *F*. *rubricaulis*, *F. rigidula*, *F*. *alliacea*, *F*. *narthex*, *F*. *latisecta*, and *F*. *lutensis*, scattered in southern and eastern Iran [16, 17].

Asafoetida is renowned due to its pharmaceutical effects in Iranian folk medicine. It has traditionally been used as an antispasmodic, antihelmintic, anticancer, carminative, antiasthmatic, antiepileptic, and analgesic agent worldwide [2, 18]. In addition, *assafoetida* has long been used as a treatment for inflammation, and several anti-inflammatory properties have been discovered. Bagheri et al. demonstrated the antinociceptive and anti-inflammatory activities of *Ferula assafoetida* in chronic and acute pain and paw oedema in mice [19, 20]. In a double-blind study, *assafoetida* improved inflammation in irritable colon disease [21]. Another study showed the effects of asafoetida for reducing and healing inflammatory markers and cartilage impairment triggered by rheumatoid arthritis in rats [22]. This evidence provides dependable reasons for assessing assafoetida's effects on reducing inflammation and identifying the possible mechanisms involved in this action. Our main objective of the research is to explore the inhibitory efficacy of ethanolic extract of *Ferula assafoetida* oleo-gum-resin or asafoetida (EEA) on TNF-*α*-stimulated human umbilical vein endothelial cells (HUVECs).

## 2. Materials and Methods

### 2.1. Materials

5-Carboxy-fluorescein diacetate N-succinimidyl ester (CFSE) was purchased from Santa Cruz (sc-214315, Santa Cruz, CA, USA). 2,7-Dichlorofluorescein diacetate (DCFH-DA) and (4,5-dimethyl-2-thiazolyl)-2,5-diphenyltetrazolium bromide (MTT) were purchased from Sigma (D6883 and M2128, respectively, Sigma-Aldrich, St. Louis, MO, USA). RiboEx solution (301-001, GeneAll), cDNA Synthesis Kit (K1621, Thermo Fisher Scientific, CA, USA), and Ficoll with a density of 1.078 g/ml were bought from G.E. Healthcare (17-5442-02, G.E. Healthcare, Chicago, IL, USA). PE-conjugated ICAM-1 and PE-isotype control were from eBiosciences (12-0549-42 and 12-4714-42, respectively, eBioscience, Thermo Fisher Scientific, CA, USA). Trypsin, penicillin, and streptomycin (P/S), foetal bovine serum (FBS), and DMEM high-glucose medium were purchased all from GIBCO (Grand Island, NY, USA). Dimethyl sulphoxide (DMSO) was purchased from Amresco (Solon, OH, USA).

### 2.2. Preparation of EEA by Maceration

*Ferula assafoetida* oleo-gum-resin was gathered from the Tabas Region (Southern Khorasan, Iran). The voucher specimen was confirmed in the Herbarium of the School of Pharmacy, University of Medical Sciences, Mashhad, Iran (voucher number: 13257). Thirty-five grams of dried *Ferula assafoetida* oleo-gum-resin was macerated in 70% ethanol and was kept in a shaker incubator (28°C, 145 rpm) for two days. Afterwards, the solution was filtrated through Whatman filter no. 4, then it was centrifuged, and the supernatant was obtained. The remaining solution was dried at 37°C, weighed, and kept at −80°C throughout the study.

### 2.3. Cell Culture and Condition

HUVECs were gifted by Dr. M.R. Ardeshiry-Lajimi (Shahid Beheshti University of Medical Sciences, Tehran, Iran). DMEM high-glucose medium enriched by 10% *v*/*v* FBS and 1% *v*/*v* penicillin-streptomycin was provided for cells to achieve the confluence at 37°C in a wet atmosphere under 5% *v*/*v* CO_2_.

### 2.4. Cell Viability Assessment

Cell viability was measured by the MTT test. HUVECs (1 × 10^4^ cells/well in 96-well plates) were saved in supplemented media for 24 h. One day later, culture media were exchanged with 200 *μ*l serum-free culture medium consisting of different concentrations of EEA (0, 31, 62, 125, 250, and 500 *μ*g/ml) and TNF-*α* (TNF-*α*) that was cloned as previously described [23] in various concentrations (0, 0.1, 1, 10, and 100 ng/ml) for 24 h. The next day, 20 *μ*l of MTT (5 mg/ml) was applied to each well and was stored at 37°C for another 4 h. Finally, the wells were emptied and filled with 100 *μ*l of DMSO to dissolve formazan crystals. The absorbance of the solubilised formazan was read out at 570 and 630 nm using Epoch Microplate Spectrophotometer (Biotek Instrument, USA). The incubated cells in the control medium were regarded as 100% viable.

### 2.5. PBMC Adhesion Assay

In order to check the potency of the TNF-*α* in the induction of ECAMs on the cell surface of HUVECs, we performed a cell adhesion assay using peripheral blood mononuclear cells (PBMC). HUVECs were seeded in a 96-well plate (1 × 10^4^ cells/well) and were incubated until reaching confluence. The cells were then stimulated with 10, 20, and 40 ng/ml of TNF-*α* for 6, 12, and 18 h, respectively. The PBMCs were isolated from peripheral blood as described elsewhere [24] and were labelled with CFSE (1 mM) for 15 minutes at 37°C. The PBMCs were centrifuged at 1200 *g* for 5 min and washed twice with PBS (1×) to remove free CFSE. The HUVECs were washed with PBS (1×), and then 50 *μ*l of labelled PBMC suspension (2 × 10^6^ cells/ml) was added to each well and incubated for another 1 h. The plate was washed with PBS (1×) to remove unattached PBMCs. Then, each well was filled with 100 *μ*l PBS and was examined with an inverted fluorescent microscope (Olympus IX70, Japan), while photos were taken using a digital camera (Olympus DP-12, Japan). After that, the cells were lysed with 0.25% Triton X-100, and the fluorescence intensity of the lysed cells was measured at the 485 nm excitation and 535 nm emissions using a fluorescence microplate reader (Cytation 3, BioTeck, USA).

The inhibitory effect of EEA on TNF-*α*-induced PBMC adhesion was evaluated on HUVECs as pretreatment with EEA at two concentrations (125 and 250 *μ*g/ml) in starved media containing 1% *v*/*v* FBS for 6 h, followed by TNF-*α* stimulation at the optimal time.

### 2.6. Intracellular ROS Assay

The effects of TNF-*α* on intracellular ROS production in HUVECs were evaluated using the DCFH-DA protocol. First, the HUVECs (2 × 10^5^ cells/well) were cultured in a 12-well plate until confluency was reached. Then, the cells were treated with 10, 20, and 40 ng/ml of TNF-*α* for 12 h. Next, the media were discarded, and the cells were washed twice with PBS (1×) and incubated with DCFH-DA (10 *μ*M) for 20 min in the incubator. Then, the wells were washed to remove unused DCFH-DA, and then they were filled with PBS (100 *μ*L/well). Next, the cells were analyzed using an inverted fluorescent microscope, and photos were taken with a digital camera. Finally, the cells were lysed with 0.25% Triton X-100, and the fluorescence intensity of the lysed cells was determined at 485 nm excitation and 535 nm emissions via a fluorescence microplate reader.

The inhibitory impact of EEA on TNF-*α*-induced ROS production was evaluated through the pretreatment of HUVECs with EEA at two concentrations (125 and 250 *μ*g/ml) in starved media for 6 h, followed by TNF-*α* stimulation at optimal time and concentration.

### 2.7. Gene Expression Assessment Using Real-Time PCR

After the treatment of HUVECs under various conditions, total RNA was extracted by the RiboEx solution. The samples were harvested with 1 ml of solution specific for RNA extraction as instructed by the manufacturer. First, total RNA concentration and its purity were quantified. Then, complementary DNA was prepared with the RevertAid First-Strand cDNA Synthesis Kit following the guidance of the producer. For real-time PCR, amplification of the E-selectin, ICAM-1, and VCAM-1 was performed on the ABI Step-One Real-Time PCR system using SYBR Green qPCR Master Mix (A325402, Ampliqon, Denmark) according to the instructions of the manufacturer. The amplification procedures included an initial denaturation for 15 min at 95°C, denaturation for 15 s at 95°C, primer annealing for 15 s at 60°C, and extension for 30 s at 72°C, with a melting curve temperature range from 60°C to 95°C. The number of PCR cycles was set at 40 for all the reactions. Primer sequences have shown in Table 1.

### 2.8. Detection of ICAM-1 through Flow Cytometry

HUVECs were first treated with or without EEA at the required concentrations for 6 h, followed by TNF-*α* stimulation (20 ng/ml) for 12 h. After treatments, HUVECs were harvested using PBS-EDTA (10 mM), washed with PBS, and resuspended in 100 *μ*l binding buffer. Samples were then subjected to 5 *μ*l pretitrated PE-conjugated ICAM-1 antibody. The related PE-labeled isotype was used as a control. The cells were washed after 30 min incubation at 4°C in the dark, and surface expressions of ICAM-1 were detected by flow cytometry (Sysmex-partec, Cube 6).

### 2.9. Liquid Chromatography-Mass Spectrometry (LC-MS) Analysis of *Ferula assafoetida* Extract

The liquid chromatography-mass spectrometry (LC-MS) analysis was performed in an Agilent 1200 series liquid chromatography coupled with Agilent 6410 triple quadrupole mass spectrometer [25, 26]. Liquid chromatography separation was performed on an Agilent Eclipse plus C18 (2.1 × 100 mm × 3.5 *μ*m) column. The flow rate was set at 0.4 ml/min, and the mobile phase consisted of (A) water+0.1% formic acid and (B) methanol+0.1% formic acid. The gradient programs were as follows: 0.0-1.0 min 10% B, 1.0-40 min from 10% to 100% (B), 40.0-42.0 min 100% (B), and 42.0-50 min from 100% to 10% (B). The mass spectra were acquired from 100 to 1000 within 50 minutes of scan time. The positive electrospray ionisation (ESI) mode was applied for the mass spectrometer. Mass feature extraction of the acquired LC-MS data and maximum detection of peaks was done using the *MZmine* analysis software package, version 2.3.

### 2.10. Statistical Analysis

All values have been evaluated with version 16.0 SPSS software (SPSS Inc., Chicago, IL, USA). The reports were provided as the mean ± S.D. for each condition of at least three independent experiments and checked for using the parametric or nonparametric *post hoc* analysis. In addition, the raw data of flow cytometry and real-time PCR were analyzed with the FSC Express 5 and Rest 2009 software, respectively. Thus, one-way variance analysis (ANOVA) and the Tukey test were carried out to investigate group variations. Statistically, significant differences have been taken into account at *p* < 0.05, 0.01, and 0.001.

## 3. Results

### 3.1. Determining Cell Viability, PBMC Adhesion, and Intracellular ROS Production of HUVECs

**Figure 1:**
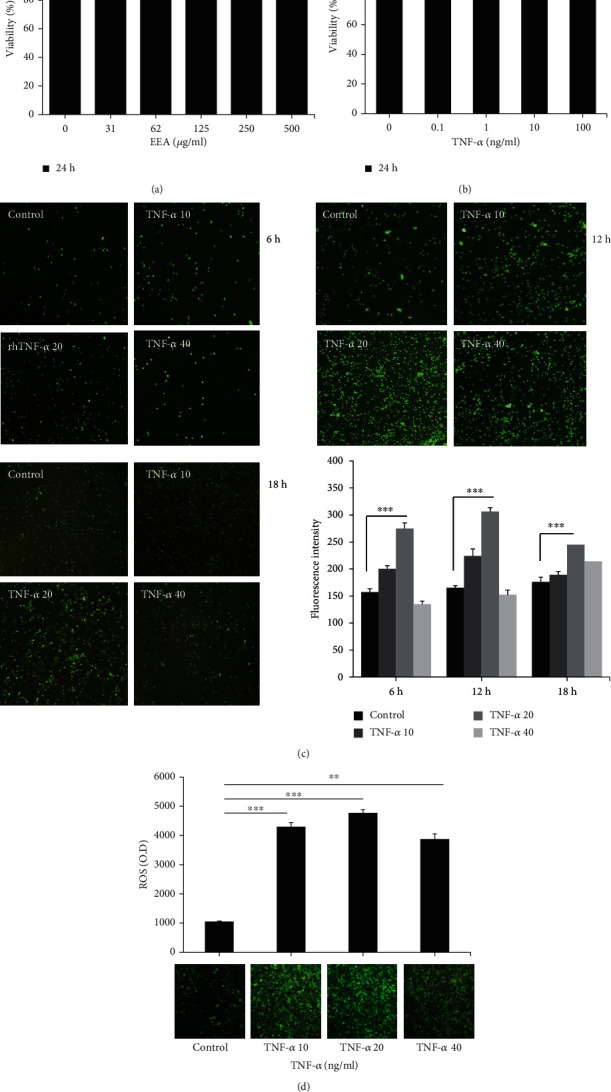
Cell viability, cell adhesion, and intracellular ROS production assay. (a) Effect of EEA on the viability of HUVECs that were treated at various EEA concentrations (0, 31, 62, 125, 250, and 500 *μ*g/ml) for 1 day. MTT test determined HUVECs' viability. The cell viability did not show significant differences up to 500 *μ*g/ml. (b) HUVECs were treated at various TNF-*α* amounts (0, 0.1, 1, 10, and 100 ng/ml) for 24 h followed by an MTT reagent to evaluate cell viability. The difference was not statistically significant in cell viability at different concentrations. (c) HUVECs were treated under different TNF-*α* levels (10, 20, and 40 ng/ml) for 6, 12, and 18 h, and a PBMC adhesion assay was carried out. (d) Cells were treated with distinct concentrations of TNF-*α* (10, 20, and 40 ng/ml) for half a day, and intracellular ROS production was assessed. Stimulation with TNF-*α* at 20 ng/ml significantly increased the PBMC adhesion and ROS production after 12 h. Every value is entered as the mean ± S.D. from at least separate triple tests. ^∗∗^*p* < 0.001 and ^∗∗∗^*p* < 0.0001 versus control. N.S.: not significant.

First of all, the cytotoxicity impact of EEA on HUVECs was tested using the MTT assay after 24 h treatment. The EEA had no significant effects on cells' viability up to 500 *μ*g/ml (Figure 1(a)). Then, we evaluated the cytotoxicity of our produced TNF-*α* on HUVECs. As shown in Figure 1(b), there was no change in cell viability of HUVECs at utilised concentration ranges from 0.1-100 ng/ml, suggesting that TNF-*α* was not directly toxic to the HUVECs.

After that, we decided to evaluate the efficacy of TNF-*α* by analyzing its ability to stimulate PBMC adhesion and intracellular ROS production in HUVECs. As illustrated in Figure 1(c), the adhesion of PBMCs to HUVECs was significantly increased by 20 ng/ml TNF-*α* in all conditions compared to their controls (^∗∗∗^*p* < 0.0001), with the most considerable rise at 12 h. Moreover, the treatment of HUVECs with indicated TNF-*α* concentrations for 12 hours showed a significant ROS production increase, which the 20 ng/ml TNF-*α* was better than other concentrations of TNF-*α* (Figure 1(d)).

### 3.2. EEA Inhibits TNF-*α*-Induced Intracellular ROS Production and Adhesion of PBMCs-HUVECs

**Figure 2:**
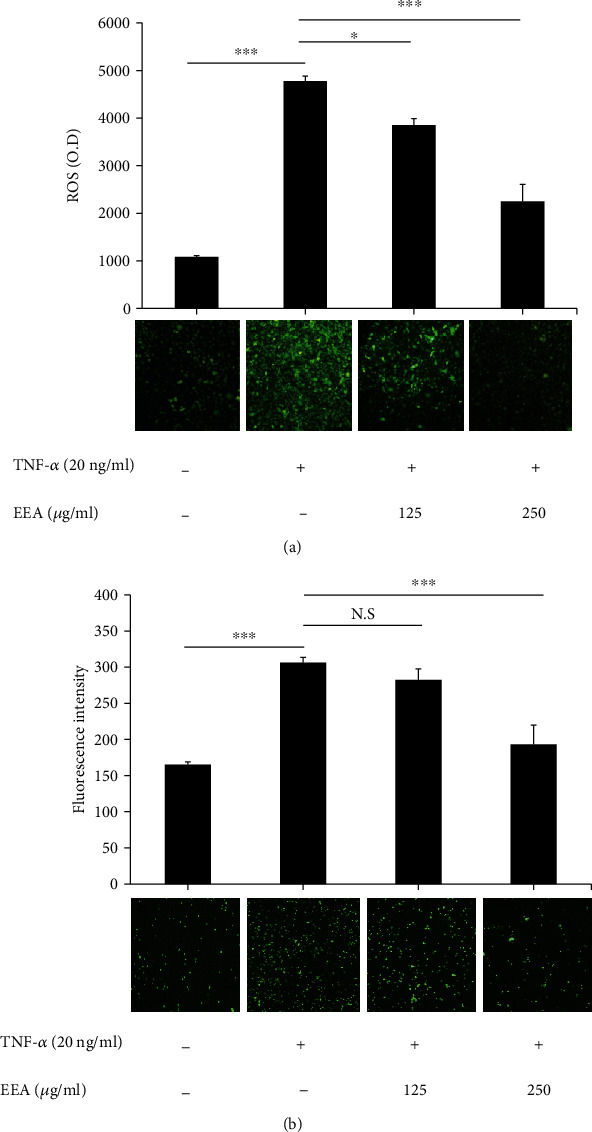
EEA reduces TNF-*α*-stimulated ROS production and PBMC adhesion to HUVECs. Target cells were incubated with two concentrations of EEA (125 and 250 *μ*g/ml) for 6 h, followed by treatment along with TNF-*α* (20 ng/ml) for 12 h. (a) DCFH-DA probes assessed ROS production. Fluorescent microscopy showed decreased ROS production in *Assafoetida* pretreatment. (b) The adhesive function of PBMCs for HUVECs was assessed. Fluorescent microscopy showed decreased adhesion in EEA pretreatment. The data reflects the mean ± SD (*N* = 3). ^∗^*p* < 0.01 and ^∗∗∗^*p* < 0.0001 opposed to control and TNF-*α*-treated groups. N. S: not significant.

We investigated whether EEA can attenuate TNF-*α*-induced ROS production. HUVECs were pretreated with EEA at defined concentrations for 6 h and then stimulated with TNF-*α* (20 ng/ml) for another 12 h. As shown in Figure 2(a), EEA reduced considerably TNF-*α*-mediated intracellular ROS production in HUVECs at both concentrations of 125 (^∗^*p* < 0.01) and 250 *μ*g/ml (^∗∗∗^*p* < 0.0001). Since the increase of intracellular ROS is an effective inducer of cell adhesion molecules, we investigated the impacts of EEA on PBMC adhesion to TNF-*α*-activated HUVECs. The findings exhibited that EEA pretreatment with 250 *μ*g/ml significantly diminished PBMCs-HUVECs adhesion (^∗∗∗^*p* < 0.01, Figure 2(b)).

### 3.3. EEA Inhibits TNF-*α*-Induced ECAM mRNA Expression in HUVECs

**Figure 3:**
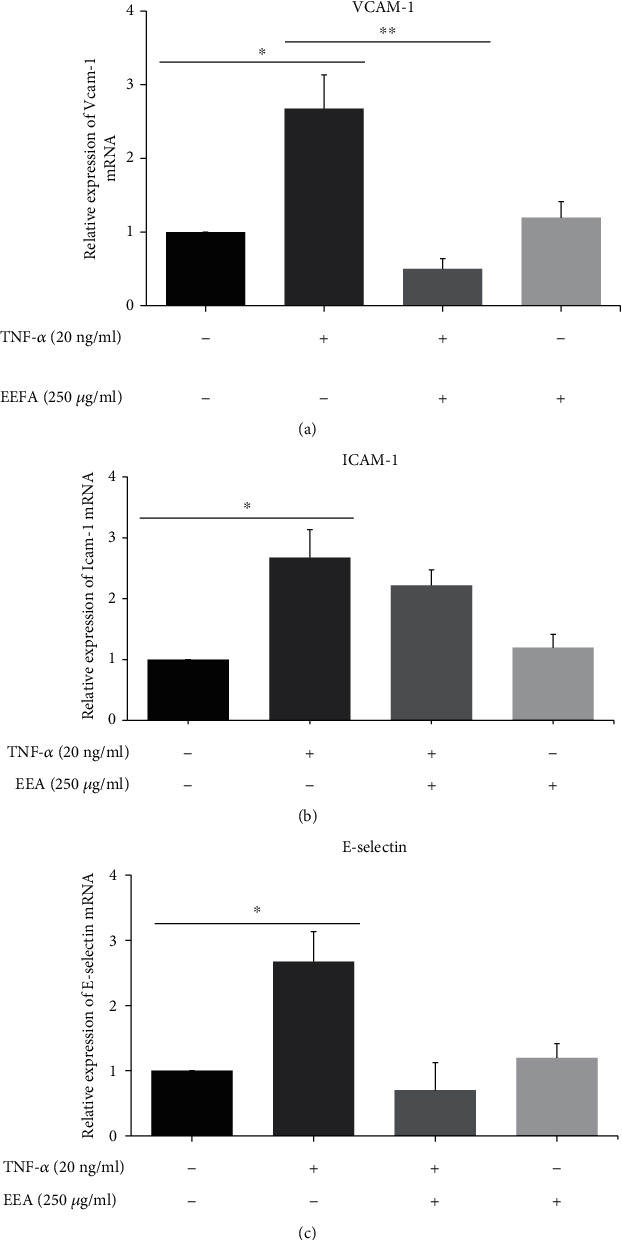
Impact of EEA on ECAM expression in HUVECs activated by TNF-*α* through real-time PCR. (a, b, c) TNF-*α* (20 ng/ml) across 12 h raises VCAM-1, ICAM-1, and E-selectin expression. TNF-*α*-induced expression of VCAM-1, ICAM-1, and E-selectin decreased with 250 *μ*g/ml of EEA for 6 h, but this reduction was not statistically meaningful except in the expression of VCAM-1. Numbers are presented as a normalised VCAM-1, ICAM-1, and E-selectin mRNA to *β*-actin mRNA, schemed in bar diagrams, and have been shown as mean ± S.D.^∗^*p* < 0.01 concerning the control group and ^∗∗^*p* < 0.001 relatives to the TNF-*α*-treated group that were calculated by *one-way ANOVA*.

Real-time PCR explorations have been done to examine the VCAM-1, ICAM-1, and E-selectin mRNA expression in activated HUVECs. Pretreatment of HUVECs with EEA (250 *μ*g/ml) for 6 h decreased VCAM-1, ICAM-1, and E-selectin gene expression parallel to the TNF-*α* stimulated group. But this decrease was just significant in VCAM-1 expression (^∗∗^*p* < 0.001) (Figure 3).

### 3.4. EEA Suppresses TNF-*α*-Induced ICAM-1 Surface Protein Expression on HUVECs

**Figure 4:**
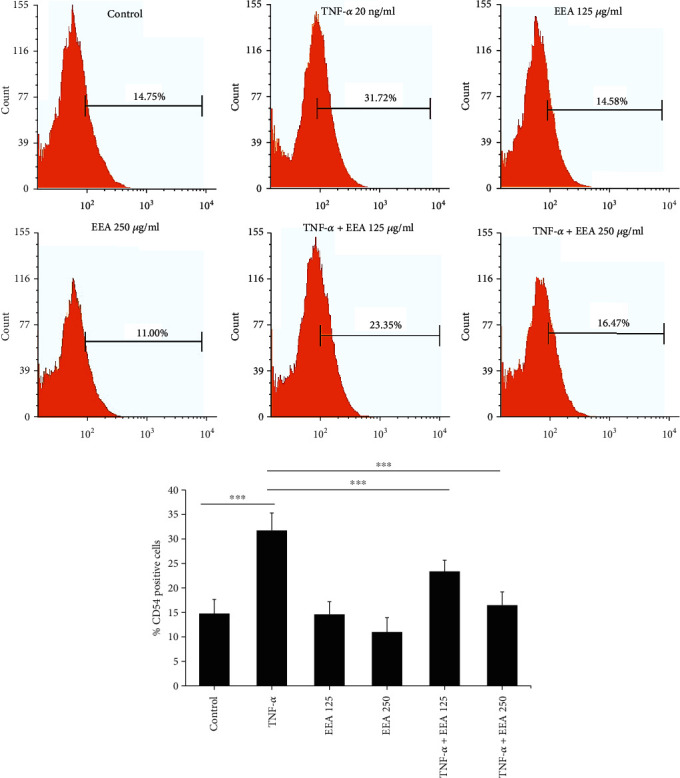
The outcome of EEA upon on surface expression of ICAM-1 in TNF-*α*-stimulated HUVECs by flow cytometry. At first, HUVECs were treated with or without *Assafoetida* (125 and 250 *μ*g/ml) for 6 h, and after that, activated with TNF-*α* (20 ng/ml) for 12 h. TNF-*α* increases ICAM-1 expression, but *Assafoetida* inhibits its expression explicitly. The parameters are reported as the mean ± S.D. for at least three separate experiments. ^∗∗∗^*p* < 0.0001 compared with the control and TNF-*α*-treated groups.

Since ICAM-1 is a crucial prerequisite for PBMCs-HUVECs adherence, we studied the effects of EEA on its protein surface distribution by flow cytometry. The findings represented that ICAM-1 protein expression (Figure 4) was deficient in unstimulated cells, and TNF-*α* (20 ng/ml) enhanced its surface aggregation. In contrast, EEA (125 and 250 *μ*g/ml) significantly reduced the expression of ICAM-1 protein in TNF-*α*-stimulation HUVECs (^∗∗∗^*p* < 0.0001) for both cases, Figure 4).

### 3.5. LC-MS Analysis of *Ferula assafoetida* Extract

**Figure 5:**
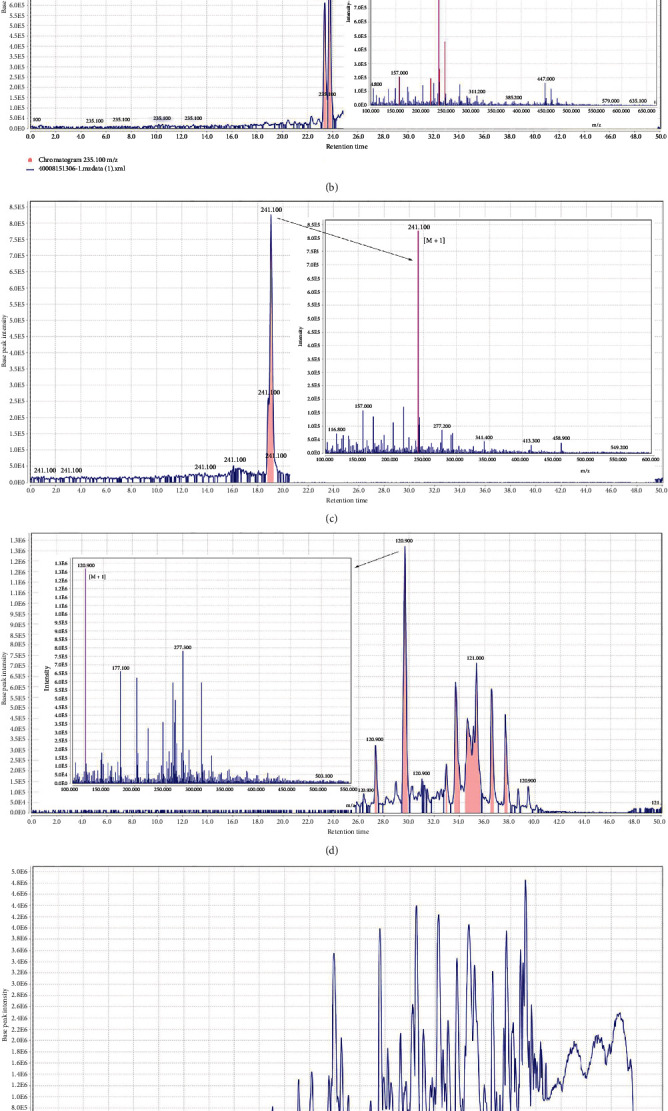
(a) The total ion chromatogram of Ferula assafoetida extract. (b) Chromatogram of umbelliprenin and corresponding mass adduct, [*M* + 1], at m/z 367.4. (c) Chromatogram of foetisulfide C and corresponding mass adduct, [*M* + 1], at m/z 241.1. (d) Chromatogram of fetidone B and corresponding mass adduct, [*M* + 1], at m/z 235.1. (e) Chromatogram of asadisulfide and corresponding mass adduct, [*M* + 1], at m/z 277.3. (f) Chromatogram of methyl-1-propenyl disulfide and corresponding mass adduct, [*M* + 1], at m/z 120.09.

Experimentally, 33 compounds were characterised in the hydroethanol extract of *Ferula assafoetida*, which is a rich source of xanthones, flavonoid-glycosides, isoflavones, and other derivatives. The identified compounds are represented in Table 2. The total ion chromatogram of *Ferula assafoetida* extract and the examples of extracted ion chromatograms from the total ion chromatogram and its related mass are shown in Figure 5. The MS spectral data were compared with the reported compounds in some previous literature. Most of the compounds detected in the *F. assafoetida* extracts have been previously reported in *Ferula* species. In addition, some coumarins (ligupersin A), sesquiterpenes (taraxacin, fetidone A, and fetidone B), and sesquiterpene coumarins (assafoetidin, umbelliprenin, assafoetidinol A, methyl galbanate, 8-acetoxy-5-hydroxyumbelliprenin, galbanic acid, epi-samarcandin, and epi-conferdione) were detected in *Ferula assafoetida* extract. Some sulfur-containing compounds (2-butyl 1-propenyl disulphide, 2-butyl 3-(methylthio)-2-propenyl disulphide, 1-methylthio-1-propene, methyl-1-propenyl disulphide, S-methylpropanethioate, dimethyl trisulfide, 2-butyl methyl disulphide, dipropyl disulphide, 2-butyl vinyl disulphide, methyl 1-(methylthio) propyl disulphide, 1-(methylthio) propyl 1-propenyl disulphide, asadisulfide, di-2-butyl trisulfide, di-2-butyl tetrasulfide, foetisulfide A, and foetisulfide C), diterpenes (7-oxocallitrisic acid, picealactone C, and 15-hydroxy-6-en-dehydroabietic acid), and acetylenes (falcarinolone) were also identified.

## 4. Discussion

Inflammation is a complex of interconnected mechanisms in reacting to various biological, chemical, and physical stimuli. Chronic inflammation can cause tissue damage and inflammatory illnesses, e.g., arthritis, atherosclerosis, and cancer. The leukocytes' movement to the inflammatory lesion and the creation of proinflammatory cytokines and chemokines are the main steps in developing chronic inflammatory diseases [27]. These events occur if E.C.s induced by proinflammatory mediators, like TNF-*α*, that stimulate ECAMs through the TNF/TNF-*α* receptor signalling cascade and ROS production [28–30]. Hence, inhibition of ROS generation and ECAM expression will be a helpful curative approach for treating vascular inflammatory disorders [31].

To the best of our knowledge, this is the first research to consider the *F. assafoetida* extract's anti-inflammatory activity on a TNF-*α* model of inflammation of the HUVECs. TNF-*α* is used in several studies with different concentrations to examine the anti-inflammatory activity of multiple compounds [32]. At this point, we induced inflammation with TNF-*α*, the most crucial proinflammatory cytokine, in a nontoxic concentration that could particularly increase PBMC adhesion levels and ECAM expression. In line with these findings, preceding experiments indicated that TNF-*α* enhanced ROS formation, monocyte adhesion, and ECAM expression [33, 34], supporting our results.

To provide a good vision for picking the working concentrations, we first carried out a cytotoxicity analysis based on a wide variety of the EEA concentrations on HUVECs. We found that the extract did not have any cytotoxicity in such cells at concentrations lower than 500 *μ*g/ml. We also observed that EEA at only 250 *μ*g/ml of EEA significantly inhibited both ROS levels and PBMC adhesion. on TNF-*α*-stimulated HUVECs, indicating the inhibitory actions of EEA on the high level of ROS and PBMC adhesion. The LC-MS analysis revealed the existence of 33 different compounds in this plant, including foetisulfide A, foetisulfide C, fetidone B, assafoetidin, umbelliprenin, and others, which may have a role in the inflammation-induced action. In this context, some studies demonstrated that luteolin and coumarin, as important constituents of the asafoetida, suppress ROS formation and reduce monocyte adhesion to TNF-*α*-stimulated HUVECs [35, 36]. Khaghanzadeh and colleagues revealed that umbelliprenin, a sesquiterpene coumarin, induces anti-inflammatory responses via an increase in the secretion of anti-inflammatory cytokines in the sera and splenocytes of mice [37]. Kohno et al. have shown that methyl galbanate derived from *Ferula szowitsiana* decreased NO generation and iNOS mRNA expression and somewhat reduced COX-2 mRNA expression in LPS/IFN-stimulated RAW264.7 cells [38]. As expected, the EEA significantly suppressed the expression of TNF-*α*-induced VCAM-1 gene and ICAM-1 protein in our research. Also, EEA decreased the ICAM-1 gene expression but was insignificant. Transcription, translation, and protein turnover are all required for cellular activity. The participation of those factors to protein levels is being debated, as transcript levels and cognate protein levels do not always correlate due to different timings, points of action, translational regulation, and protein degradation regulation, all of which are required in controlling steady-state protein abundances [39]. Breakdown of mRNA includes transcript length, ribosome density, biased codon use, and G.C. content of the third spot in codons, enhancing protein burst creation [40, 41].

A study in 2013 by Ahmadvand et al. showed that asafoetida is rich in natural antioxidant compounds such as eremophilene and *δ*-cadinene that inhibit monocytes-endothelial cell adhesion through suppressing VCAM-1 expression [19]. Furthermore, it was found that *assafoetida* contains different compounds, such as phenolic compounds, flavonoids, and compounds containing sulfur, diterpene, and sesquiterpenes [42, 43]. In 1995, Gerritsen et al. showed that some flavonoid compounds declined the expression of ICAM-1, VCAM-1, and E-selectin genes and decreased their surface expression on TNF-*α*-induced HUVECs [44]. Furthermore, in a study by Motai (2004), sesquiterpene coumarins inhibit NO development and inducible NO synthase expression of LPS-induced murine macrophage-like cell line (RAW 264.7), suggesting the anti-inflammatory effect of *Ferula fukanensis* [45].

Moreover, several studies carried out in 2004 showed that various kinds of natural antioxidant compounds, including flavonoids and polyphenolic compounds, inhibit monocyte adhesion in E.C.s [46, 47]. Therefore, according to all evidence, we hypothesised that *F. assafoetida* and its compounds might exhibit anti-inflammatory effects. Nevertheless, more experiments are necessary to identify the effects of the components of *F. assafoetida* and their anti-inflammatory activity in ex vivo and *in vivo* studies.

## 5. Conclusion

Since asafoetida has strong anti-inflammatory properties mentioned in various studies, we assessed EEA's possible anti-inflammatory molecular mechanisms. TNF-*α* can trigger kinase cascade pathways in the cell and induces NF-*κ*B in the nucleus, causing transcription and expression of adhesion genes on the cell surface. EEA via decreasing ROS generation, VCAM-1 gene expression, ICAM-1 protein expression, and leukocyte adhesion might inhibit inflammation in HUVECs. This research offers substantial proof that supports the therapeutic value of asafoetida from *Ferula assafoetida* is a new applicant for the prevention and cure of chronic inflammatory diseases, such as atherosclerotic diseases, in humans. However, further work is required to explain these promising features.

## Figures and Tables

**Table 1:** Primer sequences.

Gene	Forward primer (5′ to 3′)	Reverse primer (5′ to 3′)	PCR product size (bp)
E-selectin	TCAAGTGTGAGCAAATTGTGAAC	ATTCTCCAGAGGACATACACTGC	174
ICAM-1	AGCTTCGTGTCCTGTATGGC	CTGGCACATTGGAGTCTGCT	96
VCAM-1	ATGTCAATGTTGCCCCCAGA	ACAGGATTTTCGGAGCAGGA	119
*β*-actin	TGGGCATCCACGAAACTAC	GATCTCCTTCTGCATCCTGT	137

PCR: polymerase chain reaction; ICAM-1: intercellular adhesion molecular 1; VCAM-1: vascular cell adhesion protein 1.

**Table 2:** Peak assignment of metabolites in the hydroethanol extract of Ferula assafoetida using LC-MS in the positive mode.

Peak no.	Compound identification	*t*_*R*_ (min)	[*M* + 1] (m/z)	Ref.
1	Assafoetidin	35.9	383.3	[16]
2	Umbelliprenin	39.4	367.4	[16]
3	Assafoetidinol A	33.0	399.3	[16]
4	Methyl galbanate	40.5	413.3	[16]
5	2-Butyl 1-propenyl disulfide	34.7	163.1	[16]
6	2-Butyl 3-(methylthio)-2-propenyl disulfide	24.3	209.0	[16]
7	1-Methylthio-1-propene	31.1	103.8	[16]
8	Methyl-1-propenyl disulfide	34.6	120.9	[16]
9	S-Methylpropanethioate	33.3	104.9	[16]
10	Dimethyl trisulfide	33.6	126.9	[16]
11	2-Butyl methyl disulfide	33.9	137.0	[16]
12	Dipropyl disulfide	29.3	151.0	[16]
13	2-Butyl vinyl disulfide	34.7	149.1	[16]
14	Methyl 1-(methylthio)propyl disulfide	29.3	169.1	[16]
15	1-(Methylthio)propyl 1-propenyl disulfide	20.8	194.9	[16]
16	Asadisulfide	22.2	277.1	[16]
17	Di-2-butyl trisulfide	33.1	211.1	[16]
18	Di-2-butyl tetrasulfide	34.4	243.2	[16]
19	Foetisulfide A	23.9	225.1	[16]
20	Foetisulfide C	19.1	241.1	[16]
21	Ligupersin A	36.5	397.3	[16]
22	8-Acetoxy-5-hydroxyumbelliprenin	34.4	441.2	[16]
23	Galbanic acid	33.0	399.3	[42]
24	7-Oxocallitristic acid	40.1	329.4	[42]
25	Picealactone C	39.9	327.4	[42]
26	15-Hydroxy-6-en-dehydroabietic acid	39.4	315.4	[42]
27	Taraxacin	34.4	243.2	[42]
28	Fetidone A	23.5	233.1	[42]
29	Fetidone B	23.8	235.1	[42]
30	Falcarinolone	31.0	259.2	[42]
31	Luteolin 7-b-D-glucopyranoside	40.8	449.3	[42]
32	Epi-samarcandin	35.7	401.2	[42]
33	Epi-conferdione	39.1	395.3	[42]

## Data Availability

The data used to support the findings of this study are available on reasonable requests.

## Abbreviations

TNF-*α*: Tumour necrosis factor-alpha
EEA: Ethanolic extract of asafoetida oleo-gum-resin
HUVECs: Human umbilical vein endothelial cells
TNFR: Tumour necrosis factor receptor
PBMCs: Peripheral blood mononuclear cells
ROS: Reactive oxygen species
VCAM-1: Vascular cell adhesion protein*-*1
ICAM-1: Intercellular adhesion molecule-1
E.C.s: Endothelial cells
ECAMs: Endothelial cell adhesion molecules
NSAIDs: Nonsteroidal anti-inflammatory drugs
NO: Nitric oxide
CFSE: 5-Carboxy-fluorescein diacetate N-succinimidyl ester
DCFH-DA: 2,7-Dichlorofluorescein diacetate
MTT: (4,5-Dimethyl-2-thiazolyl)-2,5-diphenyltetrazolium bromide
FBS: Foetal bovine serum
DMSO: Dimethyl sulphoxide.
